# INDIGENOUS EXPLANATORY MODELS OF DEMENTIA: RESULTS FROM HEALTHY OLDER ADULTS IN NORTH AMERICA

**DOI:** 10.1093/geroni/igae098.0892

**Published:** 2024-12-31

**Authors:** Kristen Jacklin, Melissa Blind, Wayne Warry, Nickolas Lambrou, Carey Gleason, Megan Zuelsdorff, Dana Ketcher

**Affiliations:** University of Minnesota Medical School, Duluth, Minnesota, United States; University of Minnesota Medical School Duluth, Duluth, Minnesota, United States; University of Minnesota Medical School Duluth, Duluth, Minnesota, United States; University of Wisconsin Madison, Madison, Wisconsin, United States; University of Wisconsin Madison, Madison, Wisconsin, United States; University of Wisconsin Madison, Madison, Wisconsin, United States; University of Minnesota Medical School Duluth, Duluth, Minnesota, United States

## Abstract

Explanatory models of illness are important concepts in understanding a person’s beliefs about an illness and the social meaning that is attributed to that illness. This project seeks to understand an explanatory model of Alzheimer’s disease and related dementias (ADRD) among healthy older adults in four diverse Indigenous communities in the US (Minnesota, Wisconsin) and Canada (Ontario). Individual interviews (n=37) were conducted with healthy older adults whose age ranged from 49-79 years (M = 65.5) and were majority female (n=23, 62.2%). Four qualitative analysts conducted team-based coding, double-coding 30% of interviews to ensure intercoder agreement Kappa of ≥.80. A priori codes developed based on Kleinman’s explanatory model resulted in seven content areas: perceptions of ADRD, causes of ADRD, naming ADRD, ADRD treatment and/or care, diagnosis of ADRD/memory concerns, ADRD prevention, and fear/worry regarding ADRD. Themes under these content areas were varied but are reflective of holistic worldviews of health including physical, mental, cultural, and spiritual impacts. For example, participants report that changes associated with dementia align with completing the circle of life, so Elders may share similar traits as infants in terms of help and support. The consequences of ADRD are felt at both interpersonal and community levels, with the loss of traditional knowledge, generational memory, and stories. The default form of ADRD care is within families, with participants reporting that people should live as long as possible at home. This study provides comprehensive information about healthy older adults’ attitudes and perceptions of ADRD in Indigenous communities.

